# NPM1-fusion proteins promote myeloid leukemogenesis through XPO1-dependent HOX activation

**DOI:** 10.1038/s41375-024-02438-w

**Published:** 2024-10-23

**Authors:** Yuko Shimosato, Keita Yamamoto, Yuhan Jia, Wenyu Zhang, Norio Shiba, Yasuhide Hayashi, Shuichi Ito, Toshio Kitamura, Susumu Goyama

**Affiliations:** 1 Division of Molecular Oncology, Department of Computational Biology and Medical Sciences, Graduate School of Frontier Sciences, The University of Tokyo, Tokyo, Japan; 2 Department of Pediatrics, Yokohama City University Hospital, Yokohama, Japan; 3 Department of Hematology/Oncology, Gunma Children’s Medical Center, Shibukawa, Japan; 4 Division of Molecular Pharmacology of Malignant Diseases, Graduate School of Pharmaceutical Sciences, The University of Tokyo, Tokyo, Japan; 5 Institute of Biomedical Research and Innovation, Foundation for Biomedical Research and Innovation at Kobe, Kobe, Japan

**Keywords:** Oncogenes, Acute myeloid leukaemia

## Abstract

Nucleophosmin (*NPM1*) is a nucleolar protein and one of the most frequently mutated genes in acute myeloid leukemia (AML). In addition to the commonly detected frameshift mutations in exon12 (NPM1c), previous studies have identified *NPM1* gene rearrangements leading to the expression of NPM1-fusion proteins in pediatric AML. However, whether the NPM1-fusions are indeed oncogenic and how the NPM1-fusions cause AML have been largely unknown. In this study, we investigated the subcellular localization and leukemogenic potential of two rare NPM1-fusion proteins, NPM1::MLF1 and NPM1::CCDC28A. NPM1::MLF1 is present in both the nucleus and cytoplasm and occasionally induces AML in the mouse transplantation assay. NPM1::CCDC28A is more localized to the cytoplasm, immortalizes mouse bone marrow cells in vitro and efficiently induces AML in vivo. Mechanistically, both NPM1-fusions bind to the *HOX* gene cluster and, like NPM1c, cause aberrant upregulation of *HOX* genes in cooperation with XPO1. The XPO1 inhibitor selinexor suppressed *HOX* activation and colony formation driven by the NPM1-fusions. *NPM1::CCDC28A* cells were also sensitive to menin inhibition. Thus, our study provides experimental evidence that both *NPM1::MLF1* and *NPM1::CCDC28A* are oncogenes with functions similar to NPM1c. Inhibition of XPO1 and menin may be a promising strategy for the NPM1-rearranged AML.

## Introduction

Acute myeloid leukemia (AML) is the second most common blood cancer in children and remains a challenging disease with suboptimal outcomes [1, 2]. AML develops as a result of a series of genetic alterations in a hematopoietic progenitor cell. Recent advances in genetic characterization have improved the understanding of the biology of pediatric AML and led to the development of novel therapeutic strategies. We have previously identified potentially disease-causing genetic alterations in nearly all pediatric AML patients enrolled in the Japanese Pediatric Leukemia/Lymphoma Study Group AML-05 trial. These include two rare NPM1 rearrangements: *NPM1::MLF1* and *NPM1::CCDC28A* [3].

NPM1 is a nucleolar protein with multiple functions in ribosome biogenesis, mRNA processing and chromatin remodeling [4, 5]. Mutations of the *NPM1* gene are the most common genetic alteration in AML, detected in approximately 30% of adult AML and 7% of pediatric AML [6, 7]. NPM1 mutations are almost exclusively found in exon 12 and usually consist of 4-bp frameshift insertions that lead to the generation of an additional nuclear export signal (NES) motif at the C-terminus and loss of the nucleolar localization signal (NLS) [8]. Consequently, NPM1-mutated AML is characterized by the aberrant cytoplasmic localization of NPM1 (NPM1c) [9]. In addition to the NPM1 mutations, several *NPM1* rearrangements, such as *NPM1::MLF1*/t(3;5)(q25;q34), *NPM1::RARA*/t(5;17)(q35;q21), *NPM1::HAUS1* and *NPM1::CCDC28A*, are associated with myeloid neoplasms [10, 11]. Most of these NPM1-fusion proteins also show cytoplasmic relocalization [12]. Since the NPM1c and NPM1-fusion proteins retain the ability to interact with wild-type NPM1, they could induce cytoplasmic relocalization of wild-type NPM1 through formation of heterodimers to disrupt its normal function. Loss of NPM1 nuclear function imposed by the NPM1c or NPM1-fusion proteins has been implicated in myeloid leukemogenesis [13].

Another unique feature of NPM1-mutated AML is the aberrant activation of *HOX* genes [14, 15]. *HOX* genes encode a highly conserved family of homeodomain-containing transcription factors that specify cell identity in early development and adult tissues [16]. Aberrant overexpression of *HOX* genes is associated with a variety of malignancies including AML [17]. Recent studies have shown that both the nuclear export of NPM1c and the upregulation of *HOX* genes depend on the interaction between NPM1c and the chromatin-bound nuclear exporter XPO1 [18–20]. The NPM1c-XPO1 interaction could be disrupted by the selective nuclear export inhibitor selinexor, which covalently binds to XPO1. Preclinical studies with selinexor have shown that XPO1 inhibition induces NPM1c chromatin release and loss of *HOX* expression, thereby inhibiting the growth of NPM1-mutated AML cells. Thus, while studies have gradually revealed the molecular pathogenesis of NPM1c AML, it remained largely unknown how NPM1-fusion proteins cause AML.

In this study, we examined the leukemogenic potential of the two rare NPM1-fusion proteins, *NPM1::MLF1* and *NPM1::CCDC28A*, that are found in pediatric AML patients. *NPM1::MLF1* and *NPM1::CCDC28A* showed modest and strong leukemogenicity, respectively, in the mouse bone marrow transformation assays. We also found the critical role of XPO1 to sustain HOX gene expression and clonogenicity in the NPM1-fusion AMLs.

## Method

### Mice

C57BL/6 J mice were purchased from SLC Japan and maintained in a specific pathogen-free environment at the University of Tokyo animal center. All animal experiments were performed according to the institutional guidelines and protocol (PA21-67) approved by the Laboratory Animal Research Center of the Institute of Medical Science at the University of Tokyo.

### Patient samples

The pediatric patients with *NPM1::MLF1* fusion or *NPM1::CCDC28A* were reported by Shiba et al. [3]. The patients’ leukemic samples were obtained from the bone marrow at the time of diagnosis. RNA extraction and cDNA synthesis were performed as previously described [3]. *NPM1::MLF1* and *NPM1::CCDC28A* were amplified by polymerase chain reaction (PCR) using the following primers:

*NPM1* Fw: GCTCGAGGCCACCATGGACTACAAGGACGACGATGACAAAGAAGATTC

GATGGACATG,

*NPM1::MLF1* Rv: GCGCGGCCGCCAGTTATTTTTTGTTGCTTTTCAC,

*NPM1::CCDC28A* Rv: TGTCAATGCCAAAAAAAATGCCATTCCAGTGAG.

PCR cycling conditions were as follows: 2 min at 94 °C for one cycle, followed by 25 cycles of 10 s at 98 °C, 30 s at 55 °C, 1 min 30 s at 68 °C, followed by a final extension of 10 min at 72 °C.

### Plasmids and retrovirus infection

Flag-tagged forms of the *NPM1::MLF1* fusion and *NPM1::CCDC28A*, wild-type *NPM1*, NPM1c, *MLL::ENL* were cloned into the pMYs-IRES-GFP retroviral vector. We generated ectopic retrovirus using Plat-E packaging cells [21] with the calcium-phosphate coprecipitation method. The mouse bone marrow cells were incubated with ectopic retrovirus for 48 h using retronectin (Takara Bio Inc., Otsu, Shiga, Japan)).

### Western blotting

HEK293T cells were transiently transfected with the indicated plasmids using polyethyleneimine (PEI). Forty-eight hours after transfection, cells were lysed with cell lysis buffer (Cell Signaling Technology, Danvers, MA, USA; #9803). Lysates were subjected to sodium dodecyl sulfate-polyacrylamide gel electrophoresis and transferred to a polyvinylidene fluoride membrane (Bio-Rad). The blot was incubated with anti-Flag (Sigma-Aldrich, catalog F1804, clone M2, 1:200) and anti-GAPDH (Cell Signaling Technology, catalog #5174, clone D16H11, 1:1000) antibodies. Signals were detected with ECL Western Blotting Substrate (Promega, Madison, WI, USA) and visualized with Amersham Imager 600 (GE Healthcare).

### Immunostaining

HEK293T cells and K562 cells were transduced with the indicated plasmids using PEI. Twenty-four hours after transduction, the cells were fixed with 4% paraformaldehyde for 15 min at room temperature. The cells were then permeabilized with 0.2% Triton X-100 for 5 min and blocked with 5% goat serum for 1 h. The HEK293T cells and K562 cells were then stained with anti-flag antibody (Fujifilm, catalog 014-22383), followed by AlexaFluor633-mouse antibody (Invitrogen, catalog A-21052. Cell nuclei were stained with DAPI (BioLegend, catalog 422801). Fluorescence images were captured with an N-SIM super-resolution microscope (Nikon) and were analyzed with NIS-Elements software (Nikon).

### Colony replating assay and bone marrow transplantation assay

Eight to ten weeks old mice were treated with 5FU (150 mg/kg, intraperitoneal administration). Bone marrow cells were harvested 4 days after 5FU injection, and were transduced with pMY-IRES-GFP vector, *NPM1::MLF1* or *NPM1::CCDC28A* in RPMI supplemented with 10% fetal bovine serum, mouse SCF 50 ng/μl (R& D, catalog 455-MC), human TPO 50 ng/μl (R& D, catalog 288-TP), mouse FLT3 50 ng/μl (R& D, catalog 427-FL) for 3 days.

For the colony replating assay, 1 × 10^4^ GFP-positive cells were sorted by FACS Aria III (BD Biosciences, San Jose, CA, USA) and plated in M3234 (STEMCELL Technologies) methylcellulose containing 10 ng/mL mouse SCF (R& D, catalog 455-MC), 10 ng/mL mouse IL-3 (R& D, catalog 403-ML), mouse IL-6 (R& D, catalog 406-ML), and 10 ng/μl GM-CSF (R& D, catalog 415-ML). For each round of plating, 1 × 10^4^ cells were plated. Colonies were counted and replated every 7 days. A colony was defined as a cluster of at least 50 cells.

For the bone marrow transplantation assay, 1 × 10^6^ GFP^+^ cells were injected into 8–12 weeks old C57BL/6 J mice after sublethal irradiation (4.5 Gy). GFP^+^ leukemia cells were harvested from bone marrows of moribund mice, and were serially transplanted into sublethally irradiated secondary recipient mice by intravenous injection.

### Flow cytometry analysis

Bone marrow cells and peripheral blood cells were obtained from leukemic mice. After removal of red blood cells using RBC lysis buffer, the cells were stained with the following antibodies: CD11b-PE-cy7 (BioLegend, 101215), B220-APC-cy7 (BioLegend, 103223), CD3-APC (BioLegend, 100235) and c-kit-PE-Cy7 (BioLegend, 105814). For analysis of mouse lineage^-^ c-Kit^+^ (LK) cells, the cells were incubated with a cocktail of biotinylated monoclonal antibodies against lineage markers (CD5, B220, CD11b, Gr-1, and Ter119). Cells were then stained with c-kit-PE-Cy7 (BioLegend, 105814), Sca1-APC (BioLegend, 108112), and Streptavidin-Brilliant Violet 605 (BioLegend, 405229) CD34-Alexa flora647 (BioLegend, 119314), CD16/32-PE (BioLegend, 101307). Propidium iodide (PI) and 4’, 6-diamidino-2-phenylindole (DAPI) were used to exclude dead cells. All data were collected using FACS Verse or FACS AriaIII (BD Biosciences, San Jose, CA, USA) and were analyzed with FlowJo software.

### Morphological analysis

Cytospin preparations were stained with May-Giemsa staining solution. Images were captured using a BX51 microscope and a DP12 camera (Olympus).

### RNA Isolation and RNA sequence

Murine bone marrow cells were transduced with vector, *NPM1::MLF1* or *NPM1::CCDC28A*, and were transplanted into sub-lethally irradiated mice. Ten weeks after transplantation, bone marrow cells were collected from mice and GFP^+^ cells were sorted by FACS AriaIII (BD Biosciences, San Jose, CA, USA). Total RNA was extracted from GFP^+^ cells using the RNeasy Mini kit (QIAGEN, 74004), and mRNA was purified from total RNA using poly‐T oligo‐attached magnetic beads. Pair‐end sequencing FASTQ files were aligned to the mouse reference genome (mm10) using HISAT2 30 on the Galaxy platform (https://usegalaxy.org). Raw gene counts were obtained from the read alignments using Subread 31 (v2.4.3) and converted to counts per million (CPM) by edgeR 32 (v3.32.1). After filtering out low‐expressed genes with CPM less than 1, all CPM values were log2 transformed to generate unsupervised clustering dendrograms and heat maps. Differential expression was analyzed with the linear model using limma 33 (v3.46.0). Genes with false discovery rate (FDR) < 0.05 adjusted by the Benjamini‐Hochberg method were considered significant differentially expressed genes (DEGs). For MSigDB gene pathway overlap analysis, DEGs with FDR < 0.05 were examined at http://www.gsea‐msigdb.org/gsea/msigdb/annotate.jsp.

### CUT&RUN assay

1 × 10^5^ *NPM1::CCDC28A* cells were collected per sample to perform the CUT&RUN assay using CUT&RUN kit (Cell Signaling, #86652). The CUT&RUN assay was performed according to the protocol provided by Cell Signaling (https://www.cellsignal.com/learn-and-support/protocols/cut-and-run-protocol) with an anti-flag antibody (FUJIFILM catalog 014-22383) and Rabbit (DA1E) mAb IgG XP® Isotype control antibody (Cell Signaling#66362) as a negative control.

### Quantitative RT-PCR

Total RNA was extracted using the RNeasy Mini kit (QIAGEN) and reverse transcribed (RT) using High-Capacity cDNA Reverse Transcription Kits (Applied Biosystems, 4368814). The cDNA was then subjected to quantitative RT-PCR using a SYBR Select Master Mix (Applied Biosystems). The sequences of the 5ʹ to 3ʹ primers used for RT-PCR in this study, from 5ʹ to 3ʹ are as follows:

mouse *Hoxa3* Forward CTCATTTTGCGGTCGCTATCC

mouse *Hoxa3* Reverse ATCCATGCCATTGTAGCCGTA

mouse *Hoxa9* Forward ACAATGCCGAGAATGAGAGC

mouse *Hoxa9* Reverse GTTCCAGCGTCTGGTGTTTT

mouse *Hoxa10* Forward CAGCCCCTTCAGAAAACAGT

mouse *Hoxa10* Reverse TCTTTGCTGTGAGCCAGTTG

mouse *Hoxa11* Forward GGCCACACTGAGGACAAGG

mouse *Hoxa11* Reverse GAACTCTCGCTCCAGCTCTC

mouse *Gapdh* Forward TTGATGGCAACAATCTCCAC,

mouse Gapdh Reverse CGTCCCGTAGACAAAATGGT

### ChIP-qPCR

Chromatin immunoprecipitation (ChIP) was performed using Simple chip kit (Cell Signaling Technology, #9002) with antibodies against Flag (Sigma-Aldrich, catalog F1804, clone M2, 1:200) according to the manufacturer’s protocol. The purified DNA was then used for qPCR using a SYBR Select Master Mix (Applied Biosystems). The sequences of the 5‘ to 3‘ primers used for ChIP-qPCR in this study are as follows:

mouse *Hoxb9* Forward CCACCGACTGGCTTCCTCGC

mouse *Hoxb9* Reverse CCAGGGGTCACACCTCCCCA

mouse *Hoxa9* Forward AGGAGTCGCTGCTTTCTGTT

mouse *Hoxa9* Reverse ATTAGAACGGGGAGGGGTAA

mouse *Hoxa10* Forward TAGATGCTTGCAGAAGGAAAGG),

mouse *Hoxa10* Reverse CCATATGGCAAGAGGCAAAGA

### Dual luciferase Assay

HEK293T cells were seeded in 24-well plates and co-transfected with 560 ng of each plasmid (pMYs-IG vector, *NPM1::MLF1*, *NPM1::CCDC28A*, wild-type *NPM1*, NPM1c or *MLL::ENL*), 3.8 ng pGL4.74 vector and 190 ng HoxA9 reporter [22] using PEI. Forty-eight hours after transfection, cells were lysed and firefly and renilla luciferase activities were measured with the Dual-Luciferase Assay System (Promega) according to the manufacturer’s protocol on a FLUOstar Optima.

### Selinexor treatment

Selinexor (KPT-330) was purchased from Selleckchem (catalog# S7252). For the colony forming assay, 1 × 10^4^ *NPM1::MLF1* or *NPM1::CCDC28A* expressing AML cells were cultured in M3234 (STEMCELL Technologies) methylcellulose containing 10 ng/μl mouse SCF (R& D, catalog 455-MC), 10 ng/μl mouse IL-3(R& D, catalog 403-ML), 10 ng/μl mouse GM-CSF (R& D, catalog 415-ML) together with the indicated concentrations of selinexor (10 nM, 25 nM, 50 nM, and 100 nM). The number of colonies was counted after seven days of treatment with selinexor.

For the luciferase assay, HEK293T cells were seeded in 24-well plates and co-transfected with 560 ng of each plasmid (pMYs-IG vector, *NPM1::MLF1*, *NPM1::CCDC28A*, wild-type *NPM1*, NPM1c), 3.8 ng pGL4.74 vector and 190 ng HoxA9 reporter using PEI. Selinexor (100 nM) was added to some cultures 24 h after transfection. Reporter activity was measured 48 h after transfection.

### Cell viability assay

Cells were plated into flat-bottomed 96-well plates at a density of 5 × 10^4^ for *NPM1::CCDC28A* cells, 1 × 10^5^ for cord blood CD34^+^ cells, or 1 × 10^5^ for mouse c-kit^+^ bone marrow cells per well. Cells were incubated for 72 h with Selinexor (Selleckchem catalog#S7252) at 0, 0.025, 0.05, 0.1, 0.25, 0.5, and 1 μM, or VTP50469 (MedChemExpress catalog# HY-114162) at 0, 0.1, 0.3, 1, 3, 10, and 30 μM. The growth inhibitory effect of the drugs was evaluated both individually and in combination. Following the treatment, 8 µL of Cell Counting Kit 8 (Dojindo Molecular Technologies) was added to each well, and the plates were incubated at 37 °C for an additional 1 h. The absorbance of each sample was then measured using a CLARIOstar Plus Microplate Reader (BMG LABTECH) at 450 nm.

### Statistical analysis

GraphPad Prism 9 was used for statistical analysis. Two-tailed unpaired *t* test was used for pairwise comparisons, and one-way ANOVA was used for multiple comparisons of significance. The *p* value (<0.05, <0.01, <0.001) is indicated by one to three asterisks (*, **, ***). *p* values higher than 0.05 were considered not significant (ns).

### Ethics approval and consent to participate

The present study was conducted in accordance with the Declaration of Helsinki and approved by the institutional review boards of Gunma Children’s Medical Center and the participating institutes and the ethical review board of the JPLSG AML-05 trial. Written informed consent was provided by all patients or their parents/guardians.

## Results

### NPM1 fusion proteins localize to the cytoplasm and promote myeloid leukemogenesis

**Fig. 1:**
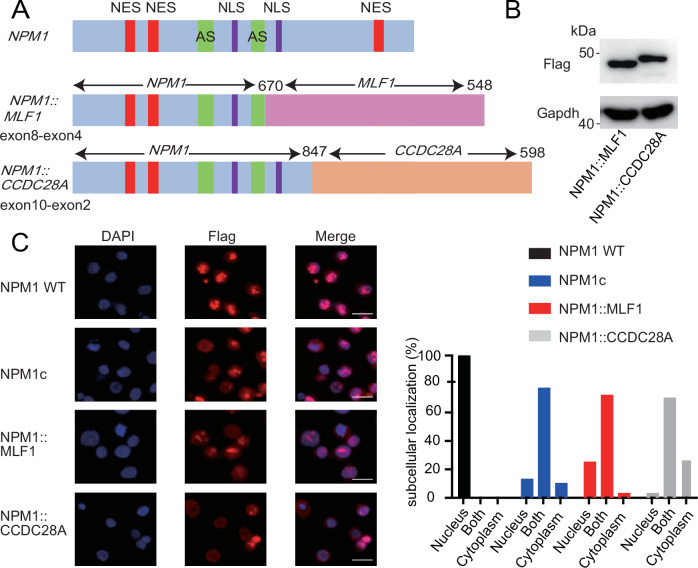
Cytoplasmic localization of the NPM1-fusion proteins. NES nuclear export signal. AS acidic domains. NLS nuclear localization signal. **A** Schematic presentation of wild-type NPM1, NPM1::MLF1, NPM1::CCDC28A. Numbers indicate positions of amino acid residues from the N-terminus. **B** HEK293T cells were transduced with the flag-tagged *NPM1::MLF1* and *NPM1::CCDC28A*. The cell lysates were stained with anti-Flag antibody. **C** K562 cells were transduced with vector, flag-tagged wild-type *NPM1*, NPM1c, *NPM1::MLF1* or *NPM1::CCDC28A*, and were then stained with anti-Flag. Cell Nuclei were visualized with DAPI. Scale bar, 20 μm. WT wild-type.

We first generated full-length *NPM1::MLF1* and *NPM1::CCDC28A* cDNAs with N-terminal Flag-tag using mRNAs isolated from AML patients harboring *NPM1::MLF1* and *NPM1::CCDC28A*. Characteristics of these patients and structures of NPM1-related proteins are shown in Table 1 and Fig. 1A, respectively. The expression of these NPM1-fusions in HEK293T cells was confirmed by Western blotting (Fig. 1B). We then examined the cellular localization of wild-type NPM1, NPM1c, NPM1::MLF1 and NPM1::CCDC28A in a transient transfection assay in HEK293T and K562 cells. As expected, wild-type NPM1 localized exclusively to the nucleolus, whereas NPM1c localized mainly to the cytoplasm in HEK293T cells and to both the nucleus and cytoplasm in K562 cells. NPM1::MLF1 was present in both the nucleus and cytoplasm, and NPM1::CCDC28A localized more to the cytoplasm in HEK293T and K562 cells. (Fig. 1C, Supplementary Figs. 1A, B). These data well agree with the previous reports [12, 23] and suggest that cytoplasmic mislocalization is a key feature of the NPM1-fusion proteins.

**Table 1:** Patient characteristics.

	Age	Sex	Karyotype	WBC	Blast(%)	FAB	SCT	Outcome	Relapse	Other somatic mutation
NPM1-MLF1	12.9	M	46,XY,t(3;5)(q24;q33)	14,400	35.6	Transformation of MDS to AML	Done	Alive	no	None
NPM1-CCDC28A	1.4	F	46,XX,add(2)(q31),add(5)(q35),add(6)(q15)[13]/46,XX[7]	16,600	40.6	M2	Done	Dead (Infection)	no	FLT3-ITD

*M* male, *F* female, *WBC* white blood cell count, *SCT* stem cell transplantation, *FAB* French-American-British (FAB) classification.

**Fig. 2:**
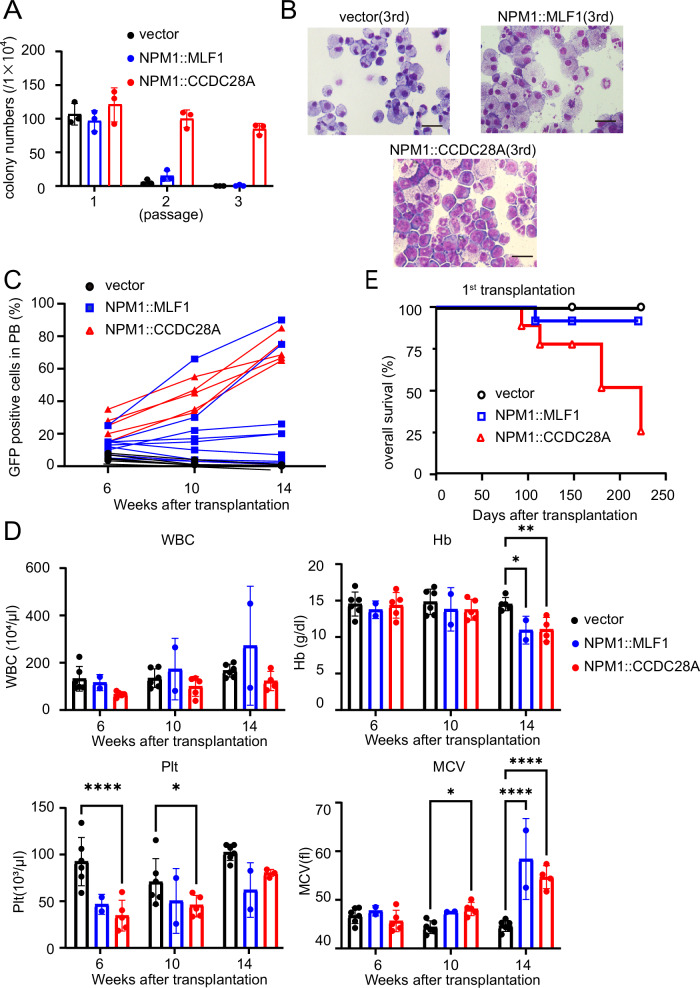
*NPM1::MLF1* and *NPM1::CCDC28A* show leukemogenic activity. **A** Colony numbers of vector, *NPM1::MLF1* or *NPM1::CCDC28A*-transduced mouse bone marrow c-Kit^+^ cells. Weekly colony count per 1 × 10^4^ replated cells are shown. Data are shown as mean ± SEM. **B** Wright-Giemsa staining of cells at the third round. All vector or *NPM1::MLF1*-transduced cells were differentiated macrophages or mastocytes, whereas *NPM1::CCDC28A*-transduced colonies contained myeloblasts. Scale bar, 20 μm. **C** Frequency of GFP^+^ cells in peripheral blood of all recipient mice bearing cells expressing vector (*n* = 6), *NPM1::MLF1* (*n* = 12) or *NPM1::CCDC28A* (*n* = 6). PB peripheral blood. **D** White blood cells, hemoglobin, platelet and mean corpuscular volume levels of the recipient mice 6, 10 or 14 weeks after transplantation are shown. Data from all recipient mice with vector-transduced cells, and mice with *NPM1::MLF1* or *NPM1::CCDC28A*-expressing cells in which the GFP^+^ cells increased after transplantation were used (vector, *n* = 6, NPM1::MLF1, *n* = 2, NPM1::CCDC28A, *n* = 6). Data are shown as mean ± SEM. One-way ANOVA was used for multiple comparisons. WBC white blood cells, Hb hemoglobin, Plt platelet, MCV mean corpuscular volume. **E** Kaplan-Meier survival curve of mice transplanted with bone marrow progenitor cells transduced with NPM1::MLF1 (*n* = 12), NPM1::CCDC28A (*n* = 6), or the vector (*n* = 6).

Next, we evaluated the leukemogenic activity of the NPM1-fusions. We transduced the vector, *NPM1::MLF1* or *NPM1::CCDC28A* into primary mouse bone marrow cells and cultured the cells in semisolid media. Vector and *NPM1::MLF1*-transduced cells did not produce colonies beyond the second passage, whereas *NPM1::CCDC28A*-transduced cells acquired extensive serial replating capacity (Fig. 2A). Furthermore, *NPM1::CCDC28A*-expressing colonies contained many myeloblasts at the third passage, whereas vector and *NPM1::MLF1*-transduced colonies consisted mainly of differentiated macrophages (Fig. 2B). Thus, *NPM1::CCDC28A* has strong activity to enhance self-renewal of mouse bone marrow progenitors in vitro.

**Fig. 3:**
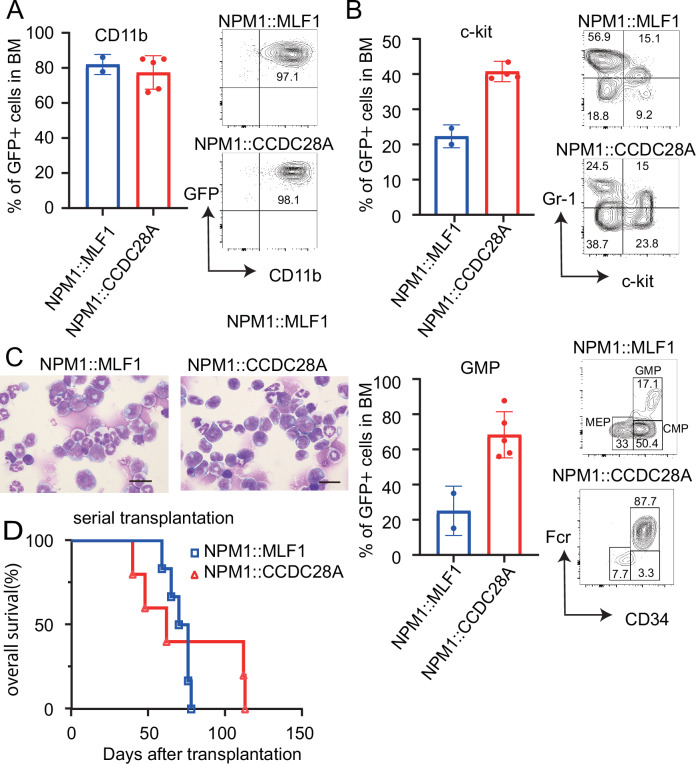
*NPM1::MLF1* and *NPM1::CCDC28A* induce the development of transplantable AML in vivo. **A–C** Bone marrow cells were collected from the moribund mice bearing *NPM1::MLF1* or *NPM1::CCDC28A*-expressing cells. BM bone marrow. **A** Representative flow cytometry profiles (right) and a bar graph showing the frequency of GFP^+^CD11b^+^ cells (left). Data are shown as means ± SEM. (NPM1::MLF1: *n* = 2, NPM1::CCDC28A: *n* = 6). **B** Representative flow cytometry profiles (right) and a bar graph showing the frequency of GFP^+^c-kit^+^ cells and GFP^+^ granulocytic-monocytic progenitors are shown. Data are shown as means ± SEM. Numbers indicate the frequency of each population. GMP granulocytic-monocytic progenitors. CMP common myeloid progenitors. MEP megakaryocyte/erythrocyte progenitors. **C** Wright-Giemsa staining of bone marrow cells collected from the recipient mice bearing *NPM1::MLF1* or *NPM1::CCDC28A*-expressing cells. Scale bar, 20 μm. **D** Kaplan-Meier survival curve of secondary recipient mice transplanted with the *NPM1::MLF1* or *NPM1::CCDC28A*-expressing cells (NPM1::MLF1, *n* = 6, NPM1::CCDC28A, *n* = 6).

We then transplanted bone marrow progenitor cells transduced with the retroviruses vector harboring *NPM1::MLF1* or *NPM1::CCDC28A* co-expressing GFP, into sub-lethally irradiated recipient mice. The *NPM1::MLF1* or *NPM1::CCDC28A-*transduced GFP^+^ cells gradually increased in the peripheral blood of some or all of the recipient mice, respectively (Fig. 2C). At 14 weeks after transplantation, the mice bearing *NPM1::MLF1* or *NPM1::CCDC28A*-expressing cells showed lower hemoglobin and higher MCV compared to the control mice (Fig. 2D). Importantly, five out of six mice transplanted with *NPM1::CCDC28A*-expressing cells and two out of twelve mice transplanted with *NPM1::MLF1*-expressing cells developed leukemia within 6 months after transplantation (Fig. 2E). Their bone marrow was dominated by GFP^+^CD11b^+^ myeloblasts (Fig. 3A), indicating that both *NPM1::MLF1* and *NPM1::CCDC28A* produced AML. *NPM1::CCDC28A*-expressing cells were enriched in a ckit^+^ granulocytic-monocytic progenitor (GMP)-like population known to contain leukemia stem cells, whereas *NPM1::MLF1*-expressing cells produced diverse cell types, including Gr1^+^ mature myeloid cells (Fig. 3B, C). Finally, we transplanted the *NPM1::MLF1*- or *NPM1::CCDC28A*-expressing AML cells into secondary recipient mice. In these serial transplantation assays, all recipient mice rapidly developed AML within 2 months after transplantation (Fig. 3D).

Taken together, we concluded that both *NPM1::MLF1* and *NPM1::CCDC28A* have leukemogenic activities. *NPM1::CCDC28A* efficiently drives AML development with a strong transforming capacity. *NPM1::MLF1* is weakly leukemogenic and likely requires additional mutations for the development of AML.

### NPM1-fusions induced upregulation of *HOX* genes and inflammation-associated genes in vivo

**Fig. 4:**
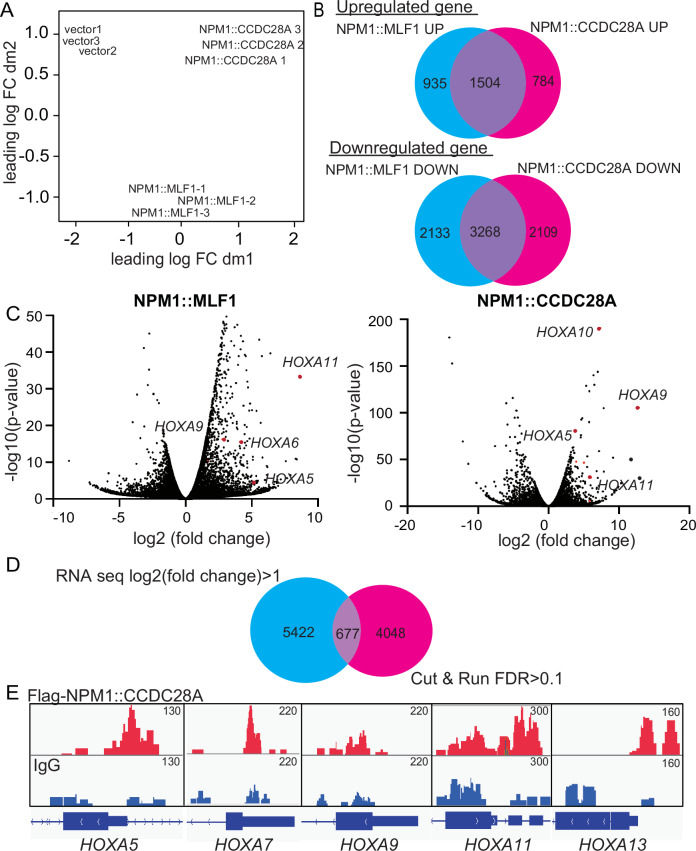
*NPM1::MLF1* and *NPM1::CCDC28A* induced specific and shared gene expression signatures. **A** Mouse bone marrow c-kit^+^ cells were transduced with vector, *NPM1::MLF1* or *NPM1::CCDC28A* and were transplanted into recipient mice. GFP^+^ bone marrow cells were harvested two months after transplantation to evaluate their gene expression profiles. Principal component analysis of GFP^+^ cells expressing vector, *NPM1::MLF1* or *NPM1::CCDC28A* is shown. FC fold change. **B** Venn diagram showing upregulated (upper panel) or downregulated (lower panel) genes in cells expressing *NPM1::MLF1* or *NPM1::CCDC28A*. **C** A volcano plot showing of up- or down-regulated genes in *NPM1::MLF1* or *NPM1::CCDC28A*-expressing cells compared to vector-transduced cells. Each dot represents one gene. **D** Venn diagram showing the overlapping genes between CUT & RUN signal (FDR > 1) and upregulated genes from RNA-seq in cells expressing *NPM1::CCDC28A*. **E** Integrative Genomic Viewer screenshots of FLAG-NPM1::CCDC28A and IgG control CUT&RUN signals around *HOX* gene clusters.

To examine the molecular changes induced by NPM1 fusions, we first examined the gene expression profiles of cells expressing vector, *NPM1::MLF1* or *NPM1::CCDC28A*. We transplanted bone marrow cells transduced with vector, *NPM1::MLF1* or *NPM1::CCDC28A* into recipient mice, and harvested GFP^+^ bone marrow cells two months after transplantation. None of the mice developed AML at this time point. *NPM1::MLF1* and *NPM1::CCDC28A*-expressing cells showed distinct gene expression profiles (Fig. 4A, Supplementary Table 1), but they also shared common features, including upregulation of *HOX* genes and inflammation-related genes (Fig. 4B, C, Supplementary Fig. 2A). We then compared the gene expression signatures of these *NPM1::MLF1* and *NPM1::CCDC28C* cells with those of other mouse AML cells driven by NPM1c, *MLL::AF9*, and *RUNX1::RUNXT1* [24–26]. The expression profiles of *NPM1::MLF1* and *NPM1::CCDC28C* cells were most similar to that of NPM1c, then to *MLL::AF9*, and were most different from that of *RUNX1::RUNXT1* (Supplementary Fig. 2B). CD34 expression was low in *NPM1::MLF1* and *NPM1::CCDC28A* cells compared to other mouse AML cells transformed by *MLL::AF9* or *SETBP1/ASXL1* mutations (which we refer to as cSAM cells), similar to that observed in NPM1c-driven AML (Supplementary Fig. 2C). In addition, we found that both *NPM1::MLF1* and *NPM1::CCDC28A* upregulated genes related to apoptosis and p53 pathway, which is likely associated with a protective proapoptotic response in hematopoietic progenitor cells (Supplementary Fig. 2D).

To identify genes that are directly regulated by NPM1-fusions, we then performed CUT & RUN analysis using *NPM1::CCDC28A* cells, which revealed *NPM1::CCDC28A* specific peaks around 4725 genes (FDR < 0.1). We integrated CUT & RUN and RNA seq analysis and identified 677 genes that are directly bound and upregulated by *NPM1::CCDC28A*, including *HOX* genes (Fig. 4D, E, Supplementary Table 2). Thus, *NPM1::MLF1* and *NPM1::CCDC28A* induced specific and shared gene expression signatures during the process of myeloid transformation. Importantly, both NPM1-fusions induced *HOX* gene upregulation like NPM1c, suggesting that they have similar functions to NPM1c.

### Selinexor inhibits colony formation and HOX gene activation induced by the NPM1-fusions

**Fig. 5:**
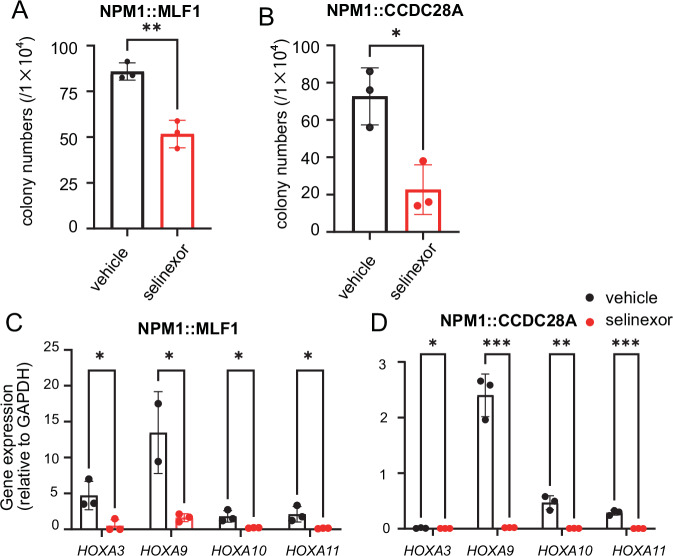
Selinexor inhibits colony formation and *HOX* gene activation induced by the NPM1-fusions. **A** Colony numbers of *NPM1::MLF1*-induced leukemia treated with either 100 nM selinexor or DMSO for 7 days. Error bars indicate the standard error (SE). Two-tailed unpaired *t* test was used for pairwise comparisons. **B** Colony numbers of *NPM1::CCDC28A*-induced leukemia treated with either 100 nM selinexor or DMSO for 7 days. Error bars indicate the standard error (SE). Two-tailed unpaired *t* test was used for pairwise comparisons. **C**, **D** *HOXA* gene levels in cells expressing *NPM1::MLF1* (**C**) or *NPM1::CCDC28A* (**D**) treated with/without 100 nM selinexor for 7 days. Data are shown as means ± SEM. Two-tailed unpaired *t* test was used for pairwise comparisons.

Previous studies have shown the critical role of XPO1 in promoting NPM1c-induced leukemogenesis by either promoting nuclear export [19] or chromatin binding to *HOX* clusters of NPM1c [18]. We therefore examined the effect of an XPO1 inhibitor selinexor on the colony forming activity of AML cells expressing *NPM1::MLF1* and *NPM1::CCDC28A*. Selinexor substantially suppressed the colony formation of *NPM1::MLF1* and *NPM1::CCDC28A*-expressing cells even at low concentrations (50–100 nM), whereas it had only a marginal effect on that of normal bone marrow progenitors (Supplementary Fig. 3A, Fig. 5A, B). In addition, selinexor (100 nM) induced downregulation of HOX genes (*HOXA3*, *HOXA9*, *HOXA10* and *HOXA11*) in *NPM1::MLF1* and *NPM1::CCDC28A* expressing cells (Fig. 5C, D). These data suggest that NPM1-fusions, like NPM1c, require XPO1 to upregulate *HOX* genes and maintain the leukemic state.

**Fig. 6:**
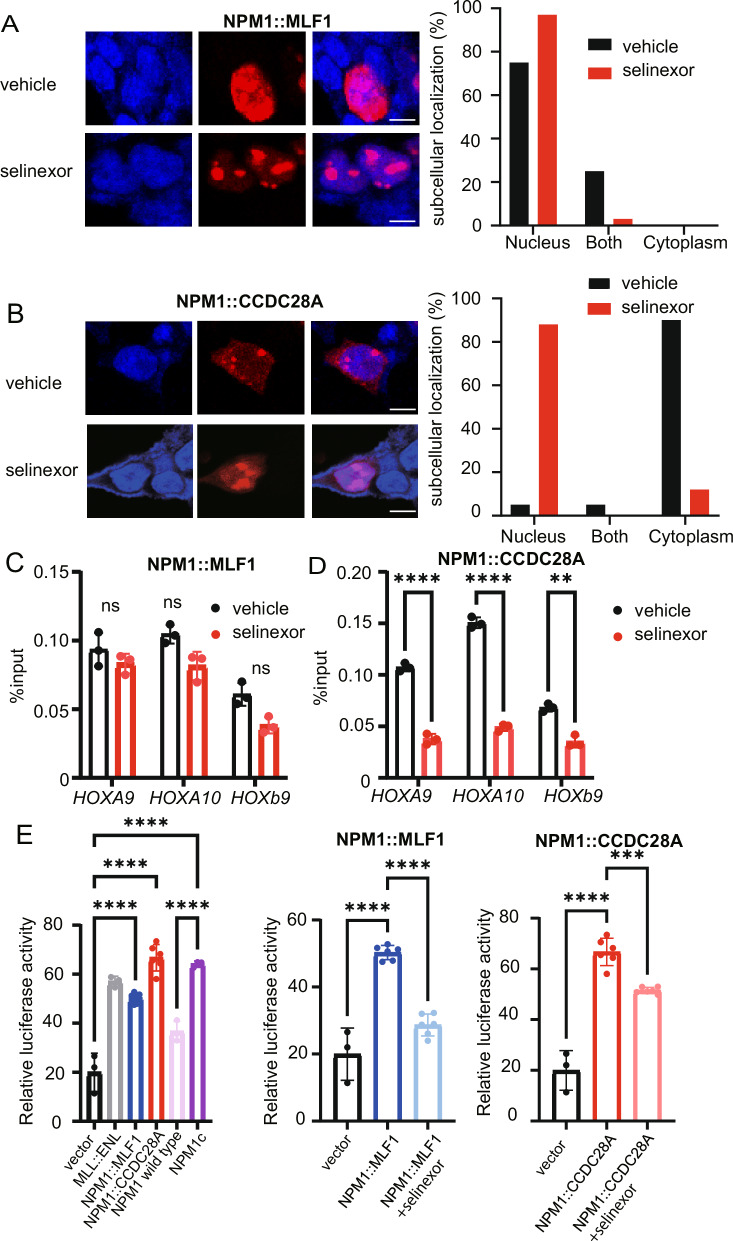
Selinexor inhibits cytoplasmic localization and chromatin binding of NPM1-fusions. 293 T cells were transfected with Flag-tagged *NPM1::MLF1* (**A**) or Flag-tagged *NPM1::CCDC28A* (**B**) and were treated with vehicle (DMSO) or Selinexor (100 nM) for 24 h. Cells were stained with anti-Flag antibody. Cell nuclei were visualized with DAPI. Representative images (left) are shown. Scale bar, 10 μm. Subcellular localization of NPM1::MLF1 and NPM1::CCDC28A was evaluated in 100 cells (right). Mouse AML cells expressing *NPM1::MLF1* (**C**) or *NPM1::CCDC28A* (**D**) were treated with 100 nM selinexor for 7 days. Binding of NPM1::MLF1 (**C**) and NPM1::CCDC28A (**D**) on the *HOX* gene cluster was assessed by ChIP-qPCR. Data are shown as mean ± SEM from triplicate wells. Two-tailed unpaired *t* test was used for pairwise comparisons. **E** 293 T cells were cotransfected with vector, *MLL::ENL*, *NPM1::MLF1*, *NPM1::CCDC28A*, wild-type *NPM1* or NPM1c together with pGL4.74 vector and HoxA9 reporter. Selinexor was added 24 h after transfection (right), and the *HOXA9* activation was measured 48 h after transfection using the Dual-Luciferase Reporter Assay System. Data are shown as means ± SEM from triplicate wells. One-way ANOVA was used for multiple comparisons.

We then assessed the effect of selinexor on the subcellular localization of NPM1::MLF1 and NPM1::CCDC28. As expected, selinexor treatment suppressed nuclear export, resulting in nuclear retention of both NPM1-fusion proteins in 293 T cells (Fig. 6A, B). Since recent studies have reported the novel role of XPO1 in recruiting NPM1c to *HOX* clusters, we next investigated whether NPM1-fusions also bind to the HOX clusters in cooperation with XPO1. We performed chromatin immunoprecipitation quantitative PCR (ChIP-qPCR) using 293 T cells transfected with vector, *NPM1::MLF1* or *NPM1::CCDC28A* in the presence or absence of selinexor. Selinexor significantly reduced the binding of NPM1::CCDC28A to the HOX clusters, and tended to reduce the binding between NPM1::MLF1 and the HOX clusters (Fig. 6C, D). To further investigate the association between NPM1-fusion proteins and HOX genes, we examined the transcriptional activity of *NPM1::MLF1*, *NPM1::CCDC28A*, NPM1c and wild-type *NPM1* using the reporter construct containing the HOXA9 promoter [22]. *MLL::ENL*, an oncogenic fusion gene known to upregulate *HOX* genes through direct binding to their promoter, was also used as a positive control [27]. The NPM1-fusion proteins as well as NPM1c significantly increased the luciferase activity of the *HOXA9* reporter as efficiently as *MLL::ENL*, whereas wild-type NPM1 showed only a modest activation of the *HOXA9* promoter. Importantly, the *HOX* activation induced by the NPM1-fusions was suppressed by the selinexor treatment (Fig. 6E). Taken together, we concluded that NPM1::MLF1 and NPM1::CCDC28A cooperate with XPO1 to bind directly to the HOX gene cluster, leading to the upregulation of HOX genes.

### Co-targeting XPO1 and menin efficiently inhibited the growth of *NPM1:CCDC28A* cells

**Fig. 7:**
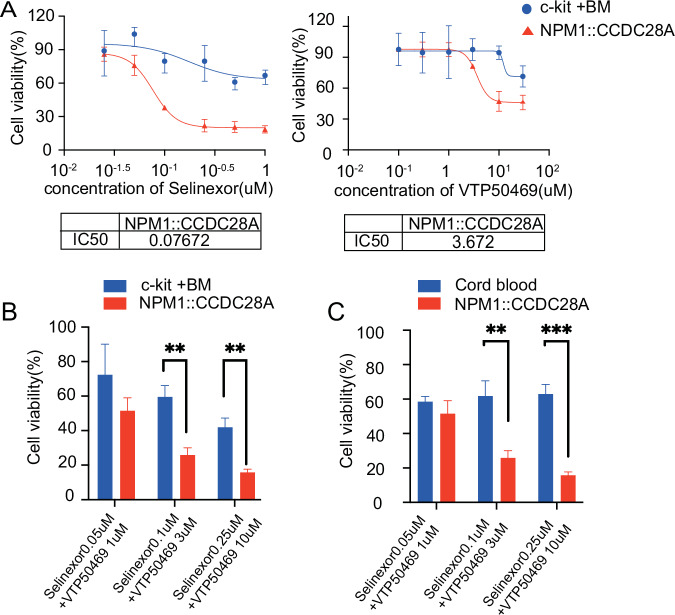
Dose-dependent effects of XPO1 and menin inhibitors on *NPM1::CCDC28A* cells. **A** *NPM1::CCDC28A* cells and mouse cKit^+^ bone marrow cells were incubated with selinexor or VTP50469 at the indicated concentration for 72 h. Cell viability was assessed using the Cell Counting Kit-8. Data are shown as means ± standard deviation (SD) from three technical replicates. *NPM1::CCDC28A* cells and mouse cKit^+^ bone marrow cells (**B**) or human cord blood CD34^+^ cells (**C**) were incubated with selinexor + VTP50469 at the indicated concentrations for 72 h. Cell viability was assessed using the Cell Counting Kit-8. Data are shown as means ± standard deviation (SD) from three technical replicates.

Recent clinical studies have shown that NPM1c AMLs are also sensitive to menin inhibitors [28]. Therefore, we next evaluated the dose-dependent effect of selinexor and a menin inhibitor (VTP50469) on *NPM1::CCDC28A* cells. VTP50469 has been shown to be effective against mouse menin [29]. Both selinexor and VTP50469 inhibited the growth of *NPM1::CCDC28A* cells in a dose-dependent manner, and *NPM1::CCDC28A* cells were more sensitive to these drugs than normal cKit^+^ bone marrow cells (Fig. 7A). Finally, we examined the combined effect of XPO1 and menin inhibition on the growth of *NPM1::CCDC28A* cells. Selinexor and VTP50469 cooperatively suppressed the growth of *NPM1::CCDC28A* cells, whereas they showed only a marginal effect on mouse cKit^+^ bone marrow cells and human cord blood CD34^+^ cells (Fig. 7B, C). These results suggest that co-targeting XPO1/menin is a promising therapeutic strategy for NPM1-fusion AMLs.

## Discussion

In this study, we demonstrated that *NPM1::MLF1* and *NPM1::CCDC28A* can induce transplantable AML in the mouse transplantation assay, providing experimental evidence that the two novel NPM1-fusions have acquired an oncogenic potential. Consistent with the previous reports [30], *NPM1::MLF1* alone was not sufficient to increase the colony-forming ability of mouse bone marrow cells in vitro and to induce AML in most recipient mice, suggesting that additional genetic alterations are required for the development of AML. Indeed, at least one pathogenic mutation (*WT1*, *FLT3-ITD*, *IDH2* or *PTPN11* mutation) has been identified in all the reported myeloid tumors harboring *NPM1::MLF1* [10]. On the other hand, *NPM1::CCDC28A* has a stronger leukemogenic activity with the ability to immortalize mouse bone marrow cells in vitro. Since *CCDC28A* also fuses with *NUP98* to form the *NUP98::CCDC28A* fusion gene in T-cell leukemia [31], it is likely that the oncogenic activity of *CCDC28A* enhances the leukemic activity of *NPM1::CCDC28A*.

Mechanistically, both NPM1::MLF1 and NPM1::CCDC28A exhibit cytoplasmic localization and induce HOX gene activation with the help of XPO1, which are very similar to the known functions of NPM1c [12, 19]. Thus, our study provides the first experimental evidence that NPM1-fusions and NPM1c share many features to promote myeloid leukemogenesis. In addition, we showed that selinexor, a selective inhibitor of XPO1 that has been used as a pan-cancer agent in preclinical and phase I clinical trials [32, 33], effectively inhibits the growth of AML cells harboring the NPM1-fusions, as it does in NPM1c AML. How the cytoplasmic NPM1 induces *HOX* gene activation in cooperation with XPO1 and how the XPO1 inhibitor selinexor suppresses leukemogenesis are currently under debate. Since XPO1 is a major transport receptor responsible for the export of proteins from the nucleus to the cytoplasm [34, 35], nuclear relocalization of NPM1c was considered to be a major mechanism of action of selinexor [19]. Indeed, we found that selinexor treatment promoted the nuclear retention of NPM1::MLF1 and NPM1::CCDC28A in 293 T cells. However, this theory could not explain how the cytoplasmic NPM1 located outside the nucleus could regulate the HOX gene expression. Instead, recent studies have proposed a novel role for XPO1 in recruiting NPM1c to the *HOXA* cluster region, leading to increased transcription of *HOX* genes [18, 20]. Indeed, we found that selinexor inhibited the binding of the NPM1-fusions to the *HOX* cluster and suppressed the activation of the *HOXA9* reporter driven by the NPM1-fusions. Thus, our data suggest that XPO1 is involved in both nuclear export and chromatin binding of the NPM1 fusion proteins. Given the strong activity of NPM1::MLF1 and NPM1::CCDC28A to directly enhance the HOXA9 reporter activity, the XPO1-mediated binding of NPM1 fusion proteins to the *HOX* gene cluster is likely to be critical for NPM1 fusion-driven myeloid leukemogenesis.

Menin inhibitors are promising new agents currently under clinical development that specifically target the HOX transcriptional program, which plays a critical role in the development of AML with *MLL* (*KMT2A)* rearrangements (MLL-r) or NPM1c. Menin inhibitors have been shown to disrupt the menin-MLL complex, induce downregulation of *HOX* genes, and promote differentiation and apoptosis of MLL-r and NPM1c AML cells. Given the potent inhibitory effect of a menin inhibitor, VTP50469, on the growth of *NPM1::CCDC28A* cells, it is likely that NPM1-fusion AMLs also depend on the menin-MLL complex to maintain the leukemogenic HOX transcriptional program. In addition, VTP50496 and Selinexor cooperatively suppress the growth of *NPM1::CCDC28A* cells, providing a rational basis for clinical testing of the XPO1/menin co-targeting approach for NPM1-fusion AML.

In summary, we demonstrated the leukemogenic activity of two novel NPM1 fusion genes, *NPM1::MLF1* and *NPM1::CCDC28A*. Both NPM1 fusions promote myeloid leukemogenesis through HOX activation with the help of XPO1. The mouse models of NPM1-fusion leukemia established in this study will be useful tools for understanding the pathogenesis of AML with NPM1 abnormalities. Our study also highlights XPO1 and menin as promising targets for the treatment of NPM1-fusion AML.

## Supplementary information

Supplemental Figure

Supplemental Table1

Supplemental Table2

## Data Availability

Sequencing data have been deposited in Gene Expression Omnibus (GSE274558, GSE274559).
