# Supplementary material for: NPM1-fusion proteins promote myeloid leukemogenesis through XPO1-dependent HOX activation
Source: Leukemia. 2024 Oct 23;39(1):75–86. doi: 10.1038/s41375-024-02438-w (PMC11717694; doi:10.1038/s41375-024-02438-w)
Supplement: Supplementary file 1 — Supplemental Figure [file 41375_2024_2438_MOESM1_ESM.pdf]

Supplemental Information

Shimosato Y et al.

NPM1-fusion proteins promote myeloid leukemogenesis through XPO1-dependent HOX activation

Supplemental Figure 1-3. ...…………………………… 2-5

Supplemental Figure1

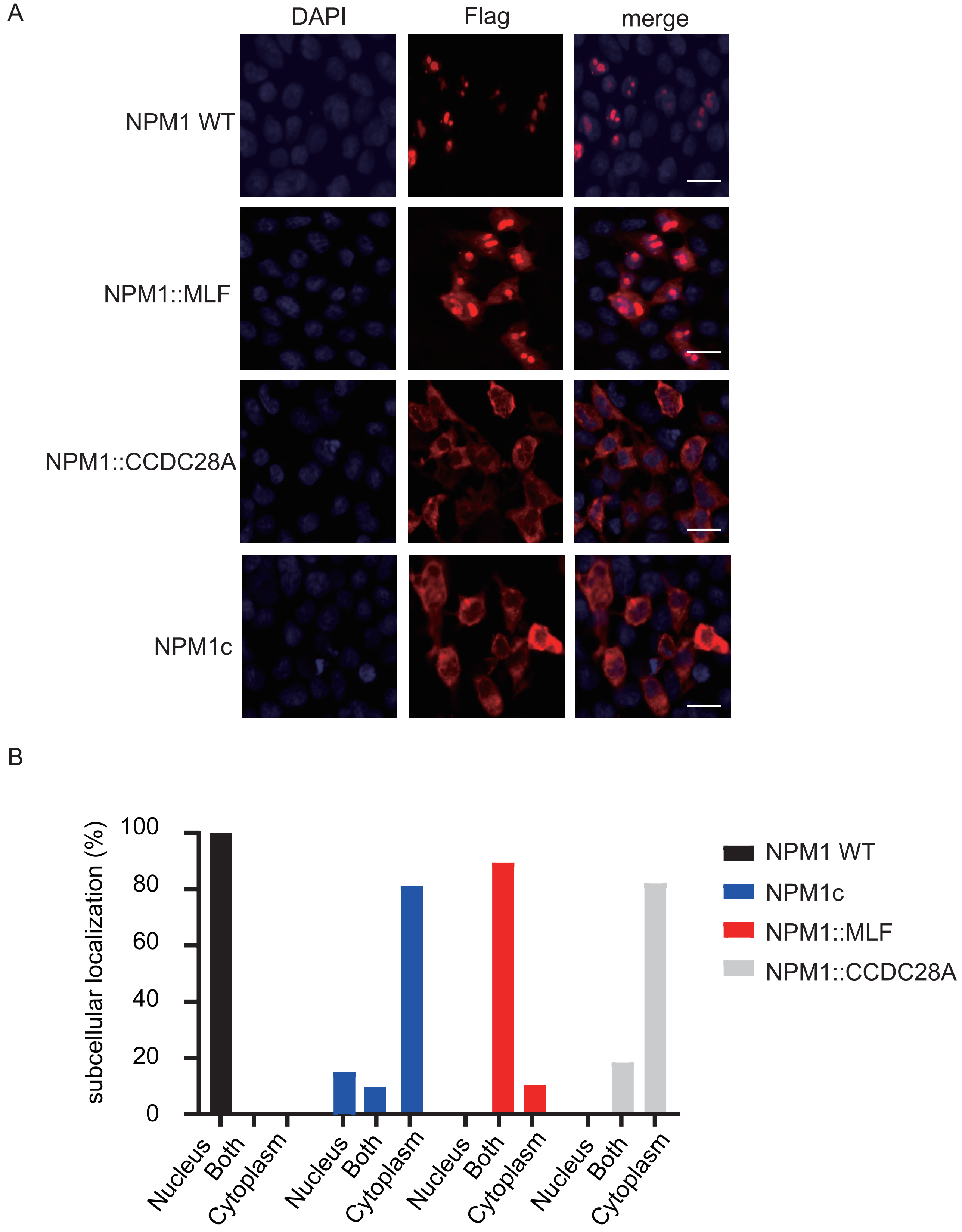

Supplemental Figure 1(Relates to Figure1)

(A) 293T cells were transfected with the flag-tagged *NPM1::MLF1* and *NPM1::CCDC28A*.

The cell lysates were stained with anti-Flag antibody.

(B) 293T cells were transfected with vector, flag-tagged wild-type (WT) *NPM1*, NPM1c, *NPM1::MLF1* or *NPM1::CCDC28A*, and were then stained with anti-Flag. Cell Nuclei were visualized with DAPI. Scale bar, 20μm.

Supplemental Figure 2

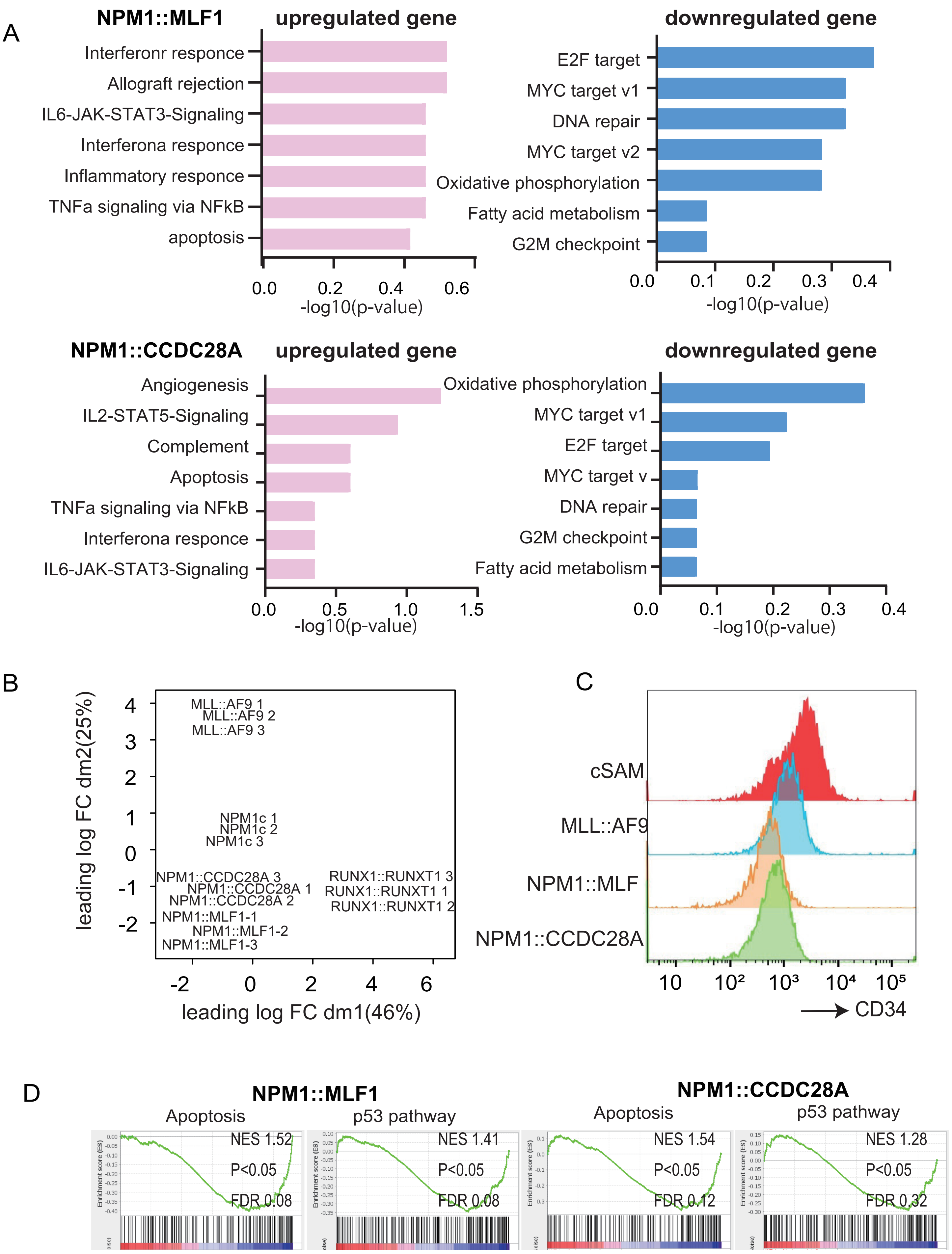

Supplemental Figure 2

Supplemental Figure 2(Relates to Figure4)

(A) Gene set enrichment analysis (GSEA) for up- or down-regulated genes in *NPM1::MLF1* and *NPM1::CCDC28A*-expressing cells using the MSigDB. The x-axis shows the p-value(−log10).

(B) Mouse bone marrow c-kit+ cells were transduced with vector, *NPM1::MLF1*, or *NPM1::CCDC28A* and were transplanted into recipient mice. GFP+ bone marrow cells were harvested two months after transplantation to evaluate their gene expression profiles. The expression data of mouse AML cells expressing *MLL::AF9*, *RUNX1::RUNXT1* (also called AML1::ETO), or NPM1c, which were deposited in GEO (GEO GSE245359 for *MLL::AF9*, GEO GSE181664 for *RUNX1::RUNXT*1, GEO GSM3717724 for NPM1c), were also used. Principal component analysis of these AML cells expressing *NPM1::MLF1*, *NPM1::CCDC28A*, NPM1c, *MLL::AF9*, or *RUNX1::RUNXT1* is shown.

(C) Representative histograms showing the expression of CD34 in mouse AML cells expressing *NPM1::MLF*, *NPM1::CCDC28A*, *MLL::AF9*, or *SETBP1* and *ASXL1* mutations (which we refer to as cSAM cells).

(D) GSEA results showing “apoptosis pathway” and “p53 pathway” signatures enriched in upregulated genes in *NPM1::MLF1* and *NPM1::CCDC28A*-expressing cells. NES, normalized enrichment score.

Supplemental Figure3

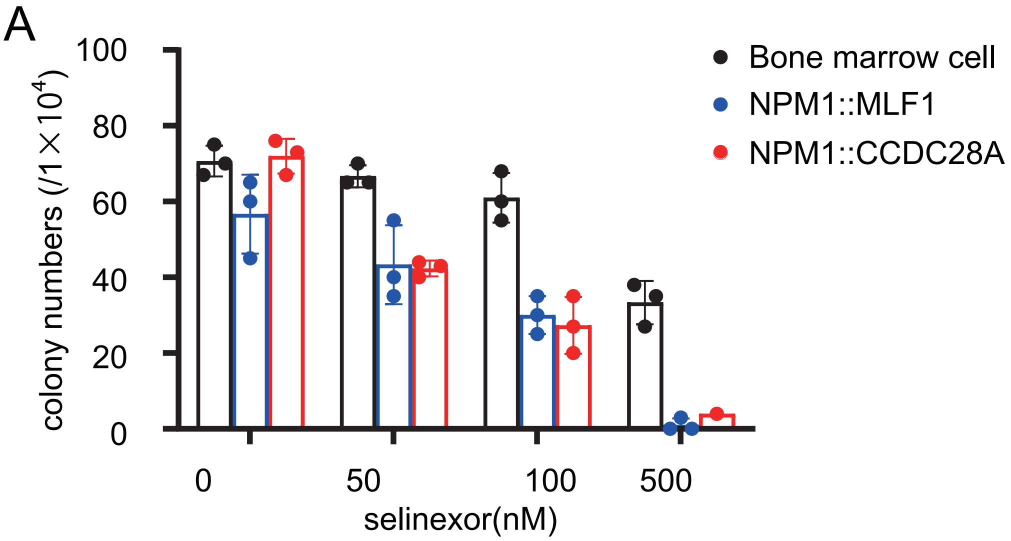

Supplemental Figure 3 (Relates to Figure5)

(A) Colony numbers of c-kit positive cells and *NPM1::MLF1*, *NPM1::CCDC28A* induced leukemia treated with increasing concentrations of selinexor for 7 days. (Selinexor 0, 50, 100, 500nM) Error bars indicate the standard error (SE). One-way ANOVA was used for multiple comparisons.
